# A Spectral Reconstruction Algorithm of Miniature Spectrometer Based on Sparse Optimization and Dictionary Learning

**DOI:** 10.3390/s18020644

**Published:** 2018-02-22

**Authors:** Shang Zhang, Yuhan Dong, Hongyan Fu, Shao-Lun Huang, Lin Zhang

**Affiliations:** 1 Department of Electronic Engineering, Tsinghua University, Beijing 100084, China; zs15@mails.tsinghua.edu.cn (S.Z.); dongyuhan@sz.tsinghua.edu.cn (Y.D.); 2 Tsinghua-Berkeley Shenzhen Institute, Tsinghua University, Shenzhen 518055, China; hyfu@sz.tsinghua.edu.cn (H.F.); shaolun.huang@sz.tsinghua.edu.cn (S.-L.H.)

**Keywords:** filter-based miniature spectrometer, spectral reconstruction, sparse optimization, dictionary learning

## Abstract

The miniaturization of spectrometer can broaden the application area of spectrometry, which has huge academic and industrial value. Among various miniaturization approaches, filter-based miniaturization is a promising implementation by utilizing broadband filters with distinct transmission functions. Mathematically, filter-based spectral reconstruction can be modeled as solving a system of linear equations. In this paper, we propose an algorithm of spectral reconstruction based on sparse optimization and dictionary learning. To verify the feasibility of the reconstruction algorithm, we design and implement a simple prototype of a filter-based miniature spectrometer. The experimental results demonstrate that sparse optimization is well applicable to spectral reconstruction whether the spectra are directly sparse or not. As for the non-directly sparse spectra, their sparsity can be enhanced by dictionary learning. In conclusion, the proposed approach has a bright application prospect in fabricating a practical miniature spectrometer.

## 1. Introduction

Spectral analysis is an elementary and indispensable approach to the qualitative and quantitative analysis of chemical materials. Thus, the spectrometer is widely used in numerous applications such as environmental monitoring, medical treatment and so on [1,2]. However, due to the equipment having sophisticated diffractive or interferometric devices such as a grating or prism, the conventional spectrometer is commonly bulky and expensive. Therefore, miniaturization of the spectrometer is a burgeoning research hotspot in both industry and academia [1,2,3,4,5,6,7,8,9,10,11,12,13,14,15,16,17,18,19,20,21]. The current commercial miniature spectrometer still utilizes a grating as its core dispersive component through the technology of micro-opto-electro-mechanical-systems (MOEMS) [3,4]. In order to reduce the size and cost of the spectrometer, various computational miniature spectrometers have been proposed and designed, in which the novel dispersive elements are employed to replace the grating [5,6,7,8,9,10,11,12,13,14,15,16,17,18,19,20,21]. For instance, photo crystal [5], linear variable optical filter [6], disordered photonic chip [7], dispersive hole array [8,9], micro interferometer array [10], liquid crystal phase retarder [11], multimode fiber [12,13], silicon multimode waveguide [14], broadband filter array [15,16,17,18,19,20] and so on are developed as the dispersive elements. Among these various miniaturization technologies, the filter-based approach shows great potential in reducing the size as well as the cost of a spectrometer, which can consequently enhance the portability and broaden the application area of spectrometry [15,16,17,18,19,20].

In the computational spectrometer, spectral reconstruction is usually modeled as solving a system of liner equations. As a result, it is necessary to use optimization algorithms to reconstruct the spectra in the computational spectrometer, which differs from the traditional direct-reading spectrometer significantly [5,6,7,8,9,10,11,12,13,14,15,16,17,18,19,20,21]. There have been several reconstruction algorithms adopted in previous studies, mainly including nonnegative least squares [5,17], least mean squares [6], simulated annealing [7,12], Tikhonov regularization [8,10,18], truncated singular value decomposition [12], direct-binary-search [21] and sparse optimization [11,13,19,20]. Several studies have demonstrated that sparse optimization performs better in terms of the reconstruction accuracy and the number of measurements [11,13,19,20]. 

**Figure 1:**
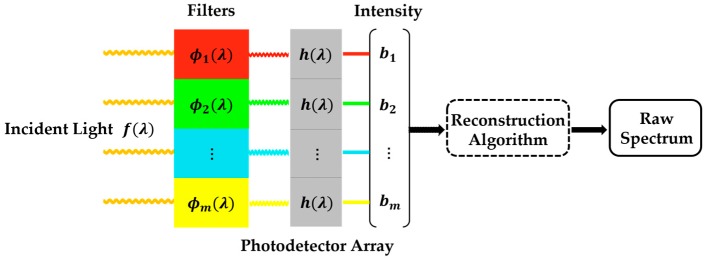
Schematic of the filter-based spectrometer.

Figure 1 presents the schematic of the filter-based miniature spectrometer. After transmitting through the filters with distinct transmission functions, the incident light is then measured by the array of photodetectors. Then the spectrum can be reconstructed from the output of the photodetector.

Ideally, the transmission functions are with a very narrow passband or similar to the delta function. Besides, different filters correspond to the specific and non-overlapping passbands, all of which jointly cover the entire operating band. In this case, the spectrum can be directly read out from the output of the photodetector, in the same way as the grating-based spectrometer. This method can be referred to as narrowband filtering. Nevertheless, it is very impractical to fabricate these ideal narrowband filters considering the craftsmanship and cost. Instead, most filters actually own the non-ideal optical filtering characteristics owing to the low-cost fabrication of broadband filters [17,18,19,20]. To be specific, their transmission functions have wide or even multiple passbands. Correspondingly, the non-ideal approach can be called broadband filtering. Unlike narrowband filtering, the detection value of broadband filtering is no longer the measured spectrum itself. Therefore, the raw spectrum needs to be reconstructed by certain signal processing algorithms [17,18,19,20]. 

Mathematically, spectral reconstruction is essentially solving a system of linear equations, where the number of equations equals that of the filters [17,18,19,20]. Given the measurement error or system noise, the system of linear equations usually needs to be overdetermined, which can be solved through the classical least squares (LS) algorithm [17,18]. Unfortunately, even when modified LS algorithms are adopted such as the nonnegative LS or Tikhonov regularized LS, reconstruction results often deviate from the true spectra owing to the ill-condition of the equations [18]. In addition, the spectral resolution is limited by the number of filters in this overdetermined setup, which implies more filters are needed to improve the resolution [17,18,19,20]. Hence, it is necessary to use advanced algorithms to reconstruct the spectra [19,20].

According to several recent studies, sparse optimization can improve reconstruction accuracy and at the same time decrease the number of needed filters [19,20]. As for the specific problem of spectral reconstruction, sparse optimization refers in particular to settling the *l*_1_-norm minimization problem, which is commonly used to solve the underdetermined system of equations [22,23,24,25]. In other words, the number of filters can be smaller than the dimensionality of the measured spectrum, which results in reducing the size and cost of the spectrometer further. However, sparse optimization highly demands the sparsity of reconstructed spectra. In general, the sparsity means that there are only a small number of nonzero components in the signal [22,23,24]. While there exist some kinds of directly sparse spectra, the vast majority of spectra are non-directly sparse and need to be represented in another transform domain.

Many natural signals are sparse in a certain transform domain [19,20], which is referred to as the dictionary in this paper. For example, an image signal has sparse representation coefficients in wavelet transformation [24,26]. Likewise, the spectra may be sparse in a certain dictionary, which needs to be discovered. Some researchers have used Gaussian kernels, Lorentzian and Secant Hyperbolic as the spectral dictionary, whose performances need to be further improved [19,20]. In the image processing, dictionary learning is an emerging method to train out the transform domain [27,28,29,30]. Inspired by this methodology, we adopt dictionary learning in this work to obtain the dictionary specific to the spectra.

In this paper, we propose an algorithm of spectral reconstruction based on sparse optimization and dictionary learning. To verify the effectiveness of the reconstruction algorithm, we design and implement a simple prototype of filter-based miniature spectrometer. The experimental results demonstrate that the *l*_1_-norm minimization is not only valid for the directly sparse spectra, but also effective for the general spectra that need transforming in the dictionary, indicating that dictionary learning can largely enhance the sparsity of general spectra. 

The rest of this paper is organized as follows. Section 2 presents the prototype of the filter-based miniature spectrometer, including the mathematical model and the experimental implementation. Section 3 details the proposed spectral reconstruction algorithm. Section 4 demonstrates experimental results for the algorithm verification. Finally, discussions and conclusions are provided in Section 5.

## 2. Modeling and Implementation of the Prototype

In this section, we aim at modeling the prototype of the filter-based miniature spectrometer and then introduce the implementation of it. 

### 2.1. System Model and Problem Formulation

As shown in Figure 1, we denote the spectrum of incident light as *f*(*λ*), a continuous function with respect to the wavelength *λ*. Then let $\phi_{i}\left(\lambda \right)\;\left(i=1,2,\ldots ,m\right)$ represent the transmittance of the *i*th filter, which is measured by the standard commercial spectrometer, and *m* stands for the number of filters. Moreover, h(λ) means the response function of the photodetector. According to the schematic, the light spectrum f(λ) is modulated respectively by different transmission functions $\phi_{i}\left(\lambda \right)$ at first and then is measured by the photodetector, the output of which is denoted as the intensity $b_{i}\in R\;\left(i=1,2,\ldots ,m\right)$ . Based on this principle, the intensity $b_{i}$ is given by

(1) $$b_{i}=\int f\left(\lambda \right)\phi_{i}\left(\lambda \right)h\left(\lambda \right)d\lambda +e_{i}\;\left(i=1,2,\ldots ,m\right)$$

where $e_{i}\in R$ represents the measurement error [17,18,19,20]. For convenience, the spectrum to be measured can be compactly denoted as the product x(λ)=f(λ)h(λ) hereafter. Therefore, the intensity $b_{i}$ is the projection of x(λ) onto the transmittance $\phi_{i}\left(\lambda \right)\;\left(i=1,2,\ldots ,m\right).$

Since the transmittance $\phi_{i}\left(\lambda \right)\;\left(i=1,2,\ldots ,m\right)$ obtained by the spectrometer can only be in the discrete form, we can approximately convert the above integral to a system of linear equations:

(2) b=Φx+e

where the intensity vector $b={\left[b_{1},\;b_{2},\ldots ,\;b_{m}\right]}^{T}\in R^{m}$ is the output of the photodetector; the sensing matrix is denoted as $\Phi ={\left[\phi_{1},\;\phi_{2},\ldots ,\;\phi_{m}\right]}^{T}\in R^{m\times n}$ , where $\phi_{i}={\left[\phi_{i}\left(\lambda_{1}\right),\;\phi_{i}\left(\lambda_{2}\right),\ldots ,\;\phi_{i}\left(\lambda_{n}\right)\right]}^{T}\in R^{n}$ is the uniform sampling of $\phi_{i}\left(\lambda \right)$ and *n* is the dimensionality of the measured spectrum; $x={\left[x\left(\lambda_{1}\right),x\left(\lambda_{2}\right),\ldots ,x\left(\lambda_{n}\right)\right]}^{T}\in R^{n}$ is the discrete form of x(λ) ; $e={\left[e_{1},e_{2},\ldots ,\;e_{m}\right]}^{T}\in R^{m}$ is the error vector corresponding to the measurement of different filters. Equation (2) is a classic inverse problem [12], and can be expanded into a more specific form:

(3) $$\left[\begin{array}{c} b_{1}\\ \begin{array}{c} b_{2}\\ \vdots \\ b_{m} \end{array} \end{array}]=[\begin{array}{cccc} \phi_{1}\left(\lambda_{1}\right) & \phi_{1}\left(\lambda_{2}\right) & \cdots & \phi_{1}\left(\lambda_{n}\right)\\ \phi_{2}\left(\lambda_{1}\right) & \phi_{2}\left(\lambda_{2}\right) & \ldots & \phi_{2}\left(\lambda_{n}\right)\\ \vdots & \vdots & \ddots & \vdots \\ \phi_{m}\left(\lambda_{1}\right) & \phi_{m}\left(\lambda_{2}\right) & \cdots & \phi_{m}\left(\lambda_{n}\right) \end{array}][\begin{array}{c} x\left(\lambda_{1}\right)\\ \begin{array}{c} x\left(\lambda_{2}\right)\\ \vdots \\ x\left(\lambda_{n}\right) \end{array} \end{array}]+[\begin{array}{c} \begin{array}{c} e_{1}\\ e_{2}\\ \vdots \end{array}\\ e_{m} \end{array}\right]$$

As mentioned earlier, in narrowband filtering, the output of the photodetector is just the discrete sampling of the spectrum x(λ) , i.e., b=x+e . Namely, Φ is the identity matrix or diagonal matrix with *m = n*. In this case, the number of filters is a key factor of limiting the spectral resolution, which implies more filters are needed to improve the resolution. 

In broadband filtering, it is necessary to adopt the optimization algorithms to solve the inverse problem (2). More precisely, there exist two situations whether *m* is larger than *n* or not. Under the condition of *m* > *n*, the LS algorithm is commonly used to solve the overdetermined system [17,18]. It is yet regrettable that reconstruction results of LS are usually unsatisfactory even though *m* is much greater than *n*. Thus, what we are actually concerned about in this paper is the underdetermined case of *m* < *n*. In this underdetermined setup, sparse optimization works very well, and will be introduced in Section 3. Furthermore, since the number of filters can be smaller than the dimensionality of spectrum, the spectral resolution will not be limited by the number of filters any more. In this way, the size and cost of the filter-based spectrometer can be reduced further.

### 2.2. Design and Implementation of the Prototype

According to Figure 1, we can design a two-dimensional filter array with distinct transmittance in different places, and then couple it to the planar photodetector array such as the charge-coupled device (CCD). With this snapshot method, we can simultaneously measure all the intensities of photodetector array, which is an efficient implementation of the filter-based spectrometer [17,18,19,20]. However, this methodology requires that the incident light should uniformly distribute on the filter array. Worse still, combining the filter array together with the photodetector will easily damage the latter during the phase of experimental verification.

To circumvent the above issues, we simplify the experimental method. Instead of fabricating the filter array, we put the filters one by one in front of the photodetector to obtain the light intensity values. The prototype demonstrated in this work is simple but effective enough to show the validity of proposed algorithm.

**Figure 2:**
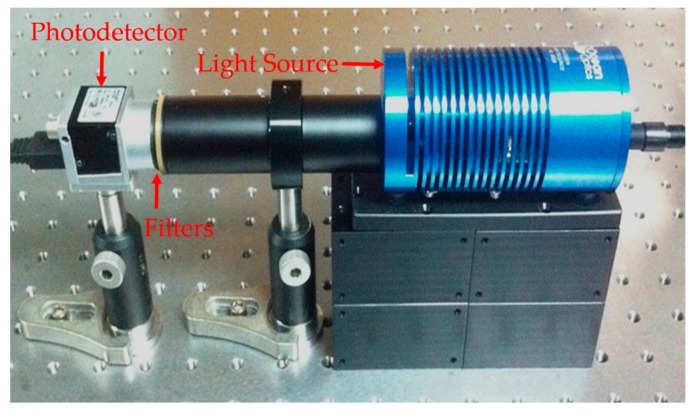
The experimental optical system.

As for the specific experimental procedure, we first preheat the light source and the photodetector to stabilize the illumination intensity and the background noise. Then we put a filter $\phi_{i}\left(\lambda \right)\;$ in front of the photodetector and measure the intensity many times to obtain the average value $b_{i}\;\left(i=1,2,\ldots ,m\right)$ . Afterwards, put another filter and repeat the above procedure. After obtaining the intensity vector *b*, we can implement the proposed algorithm to reconstruct the spectrum. The experimental optical system is shown in Figure 2. 

**Figure 3:**
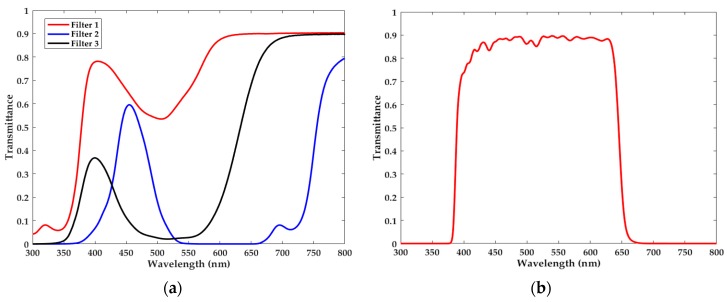
Transmission functions of (**a**) some filters and (**b**) the cut-off filter.

In this work, 210 filters with various colors are used during the experiments. In addition the transmission functions of them, which are all depicted in Appendix A, are measured by the commercial spectrometer with the sampling interval of 0.5 nm. Besides, in the experimental optical system we use a cut-off filter to acquire our concerned visible band. The transmission functions of some filters and the cut-off filter are displayed in Figure 3.

Moreover, it is a potential realization of hyperspectral imaging that we combine the methods of Figure 1 and Figure 2 together. To be specific, we can just put a rotational filter array in front of the photodetector of a camera and then take snapshots of a stationary object with the rotation of the filter array. Using the reconstruction algorithm proposed in this work, we may design a practical imaging spectrometer with small size and low cost in our future work.

## 3. Proposed Algorithm of Spectral Reconstruction

The above contents have comprehensively introduced the mathematical model and implementation details of the prototype. In this section, we elaborate on the specific algorithm of spectral reconstruction based on sparse optimization and dictionary learning.

### 3.1. Sparse Optimization

The advanced sparse optimization is well suitable for solving the noise-corrupted underdetermined system of linear equations [22,23,31]. In order to solve (2), we can simply optimize the following *l*_0_-norm minimization problem on condition that the spectrum x is sparse:

(4) $$\min_{x}\;{\left\|x\right\|}_{0}\;s.t.\;{\left\|\Phi x-b\right\|}_{2}\leq \varepsilon$$

where *l*_0_-norm ${\left\|x\right\|}_{0}$ is the number of nonzero elements of x , and *l*_2_-norm is the Euclidean norm. Besides ε is a positive constant representing the noise level. 

However, the *l*_0_-norm minimization (4) is a NP-hard problem [22,23,31], which is typically replaced by the following convex form,

(5) $$\min_{x}\;{\left\|x\right\|}_{1}\;s.t.\;{\left\|\Phi x-b\right\|}_{2}\leq \varepsilon$$

where *l*_1_-norm is defined as ${\left\|x\right\|}_{1}=\sum_{j=1}^{n}\left|x\left(\lambda_{j}\right)\right|$ . Some other versions of the *l*_1_-norm minimization to solve (2) are given in Appendix B. Herein, only if the spectrum x is sparse can we get a good approximate solution of (2) by solving (5). For example, the narrowband spectra are directly sparse. 

In practice, however, numerous spectra are not directly sparse in nature. Therefore, we need to transform the non-directly sparse spectrum x into another domain Ψ to have its sparse representation s , i.e., x=Ψs where s is a sparse vector [22,23,31]. Consequently, the original optimization problem of (5) is converted to the following form,

(6) $$\min_{s}\;{\left\|s\right\|}_{1}\;s.t.\;{\left\|\Phi \Psi s-b\right\|}_{2}\leq \varepsilon$$

After obtaining the optimal solution $s^{*}$ of (6), we can calculate the measured spectrum by $\hat{x}=\Psi s^{*}$ where $\hat{x}$ is the reconstruction of the raw spectrum x by sparse optimization.

### 3.2. Dictionary Learning

As for the non-directly sparse spectra, how to find or design a proper transform domain Ψ is still a problem to be solved. Some studies have used Gaussian kernels, Lorentzian and Secant Hyperbolic as the transform domain, which can preserve the smooth property of spectra [19,20]. Nevertheless, these approaches are tentative and not generally applicable.

In the image processing, the dictionary learning is an emerging method to train out the transform domain [27,28,29,30], which is referred to as the dictionary in this paper. Inspired by this methodology, we adopt dictionary learning in this work to obtain the spectral dictionary, which can enhance the sparsity of general spectra and then reinforce the robustness to noise. Several related studies have detailed the training process [26,27,28,29,30]. To be specific, we collect lots of spectra as the training set $T=\left[t_{1},\;t_{2},\ldots ,\;t_{p}\right]\in R^{n\times p}$ , in which $t_{i}\in R^{n}\;\left(i=1,2,\ldots ,p\right)$ is a spectrum. Then we use dictionary learning to train out the dictionary $\Psi =\left[\psi_{1},\;\psi_{2},\ldots ,\;\psi_{k}\right]\in R^{n\times k}$ such that T≈ΨD , where $D=\left[d_{1},\;d_{2},\ldots ,\;d_{p}\right]\in R^{k\times p}$ is the sparse representation matrix. Each column $\psi_{j}\in R^{n}\;\left(j=1,2,\ldots ,k\right)$ of Ψ is called an atom, and each column $d_{i}\in R^{k}\;\left(i=1,2,\ldots ,p\right)$ of D is the sparse vector representing the corresponding column $t_{i}\in \in R^{n}\;\left(i=1,2,\ldots ,p\right)$ of T in the dictionary Ψ. Herein *n* is the dimensionality of the spectrum, *p* is the total number of the spectra to be trained and *k* is the number of atoms.

Mathematically, dictionary learning can be formulated as the following optimization problem,

(7) $$\min_{\Psi ,D}\;{\left\|T-\Psi D\right\|}_{F}^{2}\;s.t.\;{\left\|d_{i}\right\|}_{0}\leq \tau \;\left(i=1,2,\ldots ,p\right)$$

where the square of Frobenius norm is defined as ${\left\|A\right\|}_{F}^{2}=\mathrm{trace}\left(A^{T}A\right)$ for the matrix A, and τ is the sparse constraint on $d_{i}\;\left(i=1,2,\ldots ,p\right)$ of the representation matrix D . There are several well-studied dictionary learning algorithms including K-SVD [27], ILS-DLA [28], RLS-DLA [29], etc. Typically, dictionary learning needs to iteratively solve the two following problems, namely the sparse approximation and dictionary update.

- Sparse Approximation Stage: keep the dictionary Ψ fixed, and then use sparse optimization above to calculate the sparse representation $d_{i}$ of $t_{i}\;\left(i=1,2,\ldots ,p\right)$ in the dictionary Ψ . That is to say, solve the inverse problem $t_{i}=\Psi d_{i}\;\left(i=1,2,\ldots ,p\right)$ by sparse optimization;
- Dictionary Update Stage: update the dictionary Ψ after obtaining a new sparse representation matrix D . There are several methods of updating the dictionary Ψ , and one can refer to the related papers for more details [26,27,28,29,30].

In our work, to obtain the training set T , we collect the transmittance of many variant chemicals in [32]. Besides, it is necessary to preprocess these spectra to make their sampling interval the same as the transmittance of the filters, i.e., 0.5 nm. In addition, the transmission functions of 210 filters are used as a part of the training set. To further increase the number of spectra to be trained, we multiply two arbitrary spectra of filters to have a new one. The physical meaning of the product is the transmittance obtained when we overlap the two filters together. At the last, the total number of the spectra to be trained is 3000, i.e., *p* = 3000. 

It is noteworthy that all aforementioned spectra of the training set are the transmission functions, which do not contain the spectral information of the light source at all. Conventionally, the spectrometer has a fixed light source with the energy spectrum of a particular shape, such as the halogen lamp. The spectral information of light source will be embedded into the detection value of the spectrometer during the measurement. In other words, the spectrum to be measured is just a modulation of the source spectrum. Hence, we need to multiply the training set of transmission functions by the spectrum of the used light source, which can be measured by the spectrometer in advance.

What is more, it should be noted that the natural optical spectra are inherently non-negative. Thus, we use the non-negative dictionary learning algorithm, which means each atom of the dictionary Ψ is a non-negative vector. Herein, the solver of non-negative K-SVD is a good option to train the non-negative dictionary [33]. Likewise, in order to reconstruct the spectra successfully, the non-negative constraint should be also added to the above formulation of sparse optimization, namely (5) and (6).

In conclusion, for the directly sparse spectra, we optimize the following problem,

(8) $$\min_{x}\;{\left\|x\right\|}_{1}\;s.t.\;{\left\|\Phi x-b\right\|}_{2}\leq \varepsilon \;x\geq 0$$

Moreover, for the non-directly sparse spectra, we turn to solve the problem below,

(9) $$\min_{s}\;{\left\|s\right\|}_{1}\;s.t.\;{\left\|\Phi \Psi s-b\right\|}_{2}\leq \varepsilon \;s\geq 0$$

where Ψ is a non-negative dictionary. There are many off-the-shelf solvers for the non-negative *l*_1_-norm minimization such as CVX [34], *l*_1_-LS [35] and TFOCS [36,37].

## 4. Results

All of the experimental results demonstrated in this section are mainly to show the feasibility and potential practicality of the prototype qualitatively. Based on these results, it can be concluded that not only does sparse optimization work well for the directly sparse spectra, but it also achieves the impressive performance when used to reconstruct the non-directly sparse spectra with dictionary learning. Furthermore, dictionary learning performs better than Gaussian kernels, which are used as the transform domain in several previous studies [19,20]. 

The main experimental instruments used in this work include a CCD camera (acA1600-20 um, Basler ace, Ahrensburg, Germany), a scientific grade spectrometer (PG2000 pro, Ideaoptics, Shanghai, China), an ultraviolet spectrophotometer (UV-2450, SHIMADZU, Kyoto, Japan), a halogen light source (HL-2000, Ocean Optics, Largo, FL, USA) and a light-emitting diode (LED) lamp. 

### 4.1. Directly Sparse Spectra

In this subsection, we aim to evaluate reconstruction quality of the continuous narrowband spectra, which are directly sparse spectra. For reconstruction of directly sparse spectra, we need to solve the *l*_1_-norm minimization problem (8).

**Figure 4:**
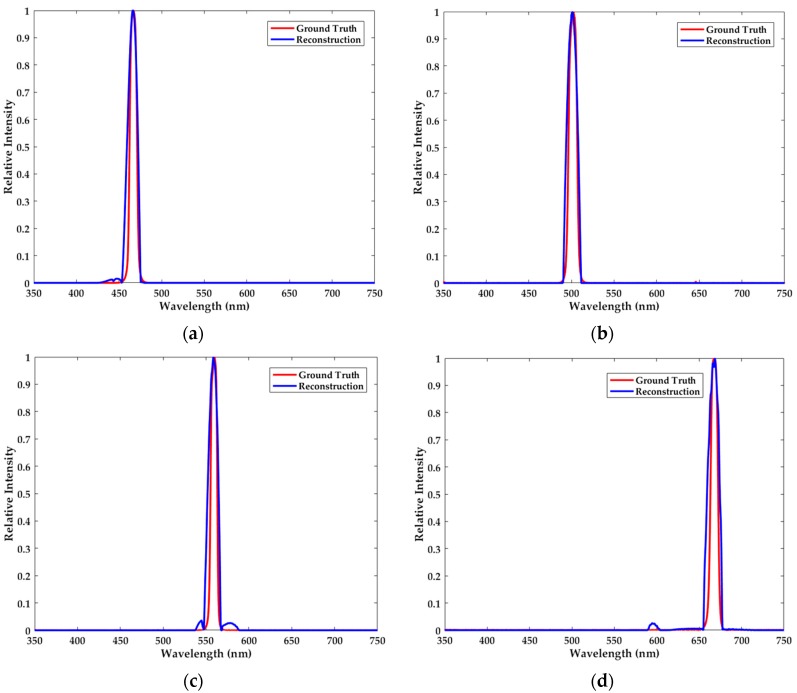
The ground truth spectra (red) and the reconstructed narrowband spectra (blue) with the crest centering at (**a**) 466.5 nm, (**b**) 501.5 nm, (**c**) 558.5 nm and (**d**) 668.5 nm. The passband and the crest of the reconstructed spectra can be accurately determined.

In this part of the experiment, we use four kinds of narrowband filters with the crest centering at 466.5 nm, 501.5 nm, 558.5 nm and 668.5 nm. Besides, their full width at half maximum (FWHM) is respectively 9 nm, 14 nm, 8 nm and 9 nm. As depicted in Figure 4, reconstruction results of the narrowband spectra are very satisfactory. It is noteworthy that there exist some slight perturbations in the neighborhood of the passband. Despite the reconstructed waveforms not completely coinciding with the ground truth spectra owing to the measurement error, the locations of the passband and crest can be accurately determined.

### 4.2. Non-Directly Sparse Spectra

Reconstruction results of non-directly sparse spectra are demonstrated in this subsection. For reconstruction of non-directly sparse spectra, we need to first adopt dictionary learning to train out the dictionary and then use the existing sparse optimization solver to solve the *l*_1_-norm minimization problem (9). 

#### 4.2.1. Halogen Lamp as the Source 

Since the halogen lamp has excellent stability and strong illumination, it is commonly used as the light source of the visible-light spectrometer in spite of its high price. To obtain the dictionary specific to the halogen lamp, we need to multiply the training set of transmission functions by the spectrum of the lamp at first and then train out the dictionary using dictionary learning. In the experiments, we first reconstruct the spectrum of the halogen lamp. Afterwards, we combine three different filters respectively together with the halogen lamp as testing samples, which are measured one after another. In this part the number of filters *m* is 210 and the operating band is from 350 nm to 700 nm with a sampling interval of 0.5 nm, i.e., *n* = 701.

**Figure 5:**
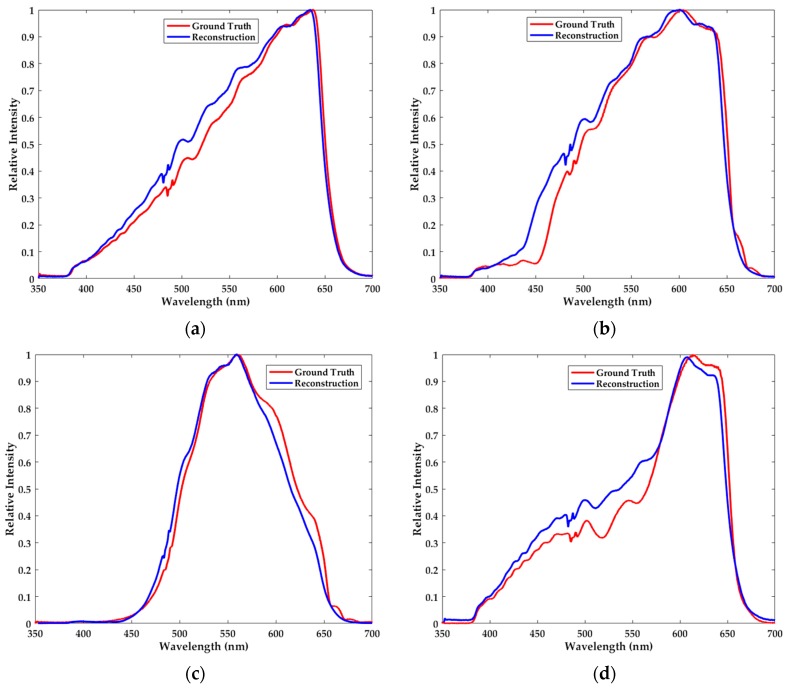
The ground truth spectra (red) and the reconstructed spectra (blue) of (**a**) the halogen lamp, (**b**–**d**) the halogen lamp with different additional filters. We combine the *l*_1_-norm minimization with the dictionary learning to reconstruct the spectra.

The results of the aforementioned experiments are listed in Figure 5. Although reconstructed spectra are somewhat discrepant from the ground truth, they share the consistent variation tendency and are similar shape as shown in Figure 5. To be specific, the crests and troughs are mostly located at the same wavelengths. 

#### 4.2.2. Light-Emitting Diode as the Source

**Figure 6:**
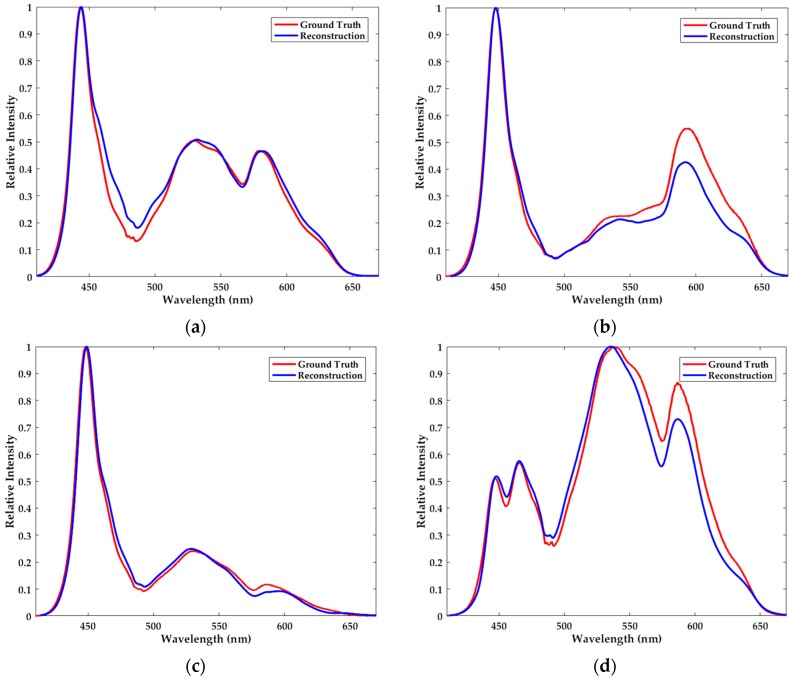
The ground truth spectra (red) and the reconstructed spectra (blue) of (**a**) LED, (**b**–**d**) LED with different additional filters. We combine the *l*_1_-norm minimization with the dictionary learning to reconstruct the spectra.

In this part of the experiment, we use a light-emitting diode (LED) as the light source. The number of used filters *m* is 210 and the operating band is from 410 nm to 670 nm with the sampling interval of 0.5 nm, i.e., *n* = 521. In general, compared to the halogen lamp, the LED is less frequently used as the light source of the commercial spectrometer. Its energy distribution along the wavelength is non-uniform. However, in our filter-based spectrometer what we need to measure is not the individual intensity at a particular wavelength but the total intensity over the spectral band. The experiments herein, whose results are displayed in Figure 6, are performed just through the same procedures as stated in the previous part. 

Likewise, the conclusions of the LED resemble those of the halogen lamp. In short, the minor deviation and the similar variation tendency coexist in the results. According to the satisfactory results, the LED may substitute the frequently used halogen lamp as the light source of the spectrometer. Consequently, the cost of the spectrometer can be further reduced by the use of inexpensive LED.

### 4.3. Comparison between Dictionary Learning and Gaussian Kernels

In this paper, we demonstrate a prototype combining sparse optimization with dictionary learning to reconstruct the spectra. Several previous studies have used Gaussian kernels as the transform domain of spectra [19,20]. Therefore, in this subsection we compare the performance of dictionary learning and Gaussian kernels in spectral reconstruction. 

We carry out the experiments respectively on spectral reconstruction of the halogen lamp and the LED. There are two main parameters that can be adjusted in the method of Gaussian kernels, namely the mean value and standard deviation. In this comparison, we let the mean value traverse in the whole operating band with the interval of 0.5 nm. Besides, we set the standard deviation of the halogen lamp and the LED to 42 and 18 respectively. These parameters have been optimized by the brute-force search. To compare reconstruction quantitatively, we calculate the relative reconstruction error *re* as follows [13],

(10) $$re=\frac{{\left\|x-\hat{x}\right\|}_{2}}{{\left\|x\right\|}_{2}}\times 100\%$$

where x is the raw spectrum and $\hat{x}$ is the reconstructed spectrum of x .

**Figure 7:**
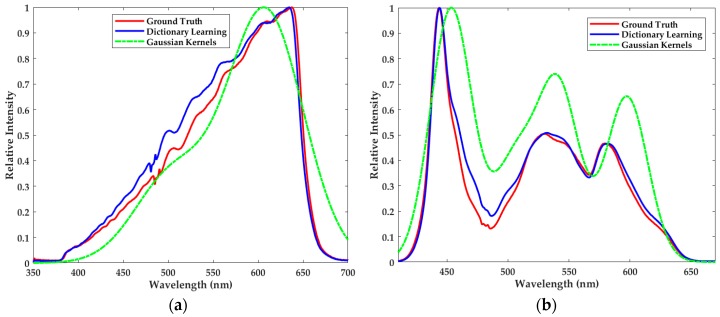
The ground truth (red) and the reconstructed spectra of (**a**) the halogen lamp and (**b**) LED using the learned dictionary (blue) and Gaussian kernels (green).

Relative reconstruction errors of dictionary learning and Gaussian kernels are respectively 5.92% and 21.09% in Figure 7a. Besides, for reconstruction of LED, the relative errors are 10.25% and 57.53% in Figure 7b. Based on these relative errors, dictionary learning performs better than Gaussian kernels. As depicted in Figure 7, the results of Gaussian kernels severely deviate from the ground truth. Worse still, it is so hard and tentative to adjust the parameters that Gaussian kernels cannot be applied in the actual spectral reconstruction. Even though we increase the number of kernels with multiple variances, there is no further improvement of reconstruction quality. While the method of Gaussian kernels can preserve the smooth feature of the spectra, it cannot capture the variation details and has little robustness to the measurement error in the experiments. According to these results, we can conclude that dictionary learning indeed contributes to finding the sparse representation of the spectra, and is beneficial for obtaining better reconstruction results when combined with sparse optimization.

### 4.4. Further Exploration

**Figure 8:**
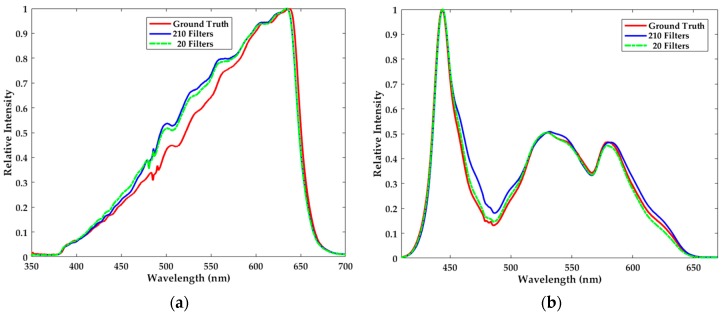
The effect of the number of filters on the reconstruction quality of (**a**) the halogen lamp and (**b**) LED.

As previously mentioned, the *l*_1_-norm minimization is adopted to solve the underdetermined system of linear equations, in which the number of filters is far smaller than the dimensionality of spectrum. The fewer the filters are, the more simply the miniature spectrometer is fabricated. Besides, the total cost will be lowered further. Herein we explore the effect of the number of filters on reconstruction quality. As shown in Figure 8, reconstruction results are even slightly improved when we bring the number of filters down from 210 to 20, which is seemingly unaccountable but in fact reasonable.

**Figure 9:**
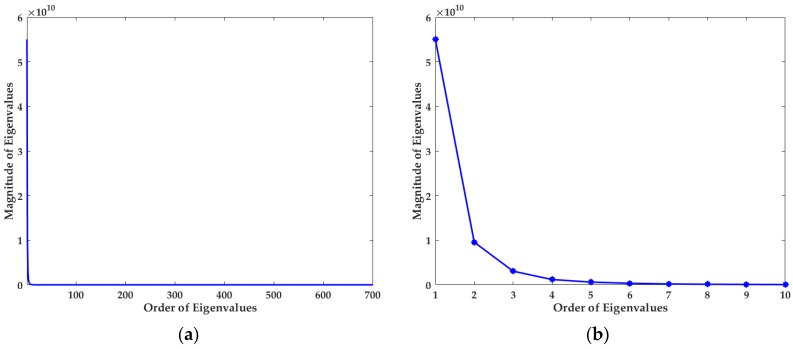
(**a**) All of the eigenvalues and (**b**) the ten largest eigenvalues of PCA.

In order to explain the results, the principle component analysis (PCA) is adopted to validate the sparsity of the spectra [38,39,40,41]. Specifically, PCA is carried out on the training set of the halogen lamp. The dimension of the spectra in the training set is 701, i.e., *n* = 701. Figure 9a plots all the eigenvalues of PCA in descending order, and the ten largest eigenvalues are shown in Figure 9b. Based on the theory of PCA, these rapidly descending eigenvalues visually indicate that the information contained in the spectra is very sparse, which is the necessary precondition of sparse optimization. Besides, according to Section 4.3, dictionary learning indeed largely enhances the sparsity of spectra and further makes the spectral reconstruction more robust to noise. Based on these facts, 20 proper filters actually may capture the vast majority of the spectral information. In addition, more filters will bring about more noises in our simplified method. For these reasons, when we bring the number of filters down from 210 to 20, it is possible to obtain better reconstruction results.

However, it should be clearly stated here that not every twenty filters out of the 210 could succeed in reconstructing the spectra as in Figure 8. That is to say, one group of 20 filters may get better reconstruction result, while the result of another group may be worse. Based on these findings, it can be inferred that fewer filters may put forward more stringent requirements on the property of the transmittance matrix, which will be further studied in future work. 

## 5. Discussion and Conclusions

Among various miniaturization technologies, the filter-based spectrometer is a promising implementation, in which the inexpensive broadband filters replace the sophisticated diffractive or interferometric optical devices used in the traditional spectrometer, such as the grating or prism. The consequent problem is how to design the proper reconstruction algorithm due to the non-ideal filters. In this paper, the spectral reconstruction is accomplished by sparse optimization and dictionary learning, which can minimize the number of filters. The results demonstrate that sparse optimization applies well to the spectral reconstruction whether the spectra are directly sparse or not. For the non-directly sparse spectra, their sparse representation is obtained by dictionary learning, which outperforms the method of Gaussian kernels even with the optimized parameters by brute-force searching. In addition, according to the reconstruction quality of LED, it may be an alternative source in the filter-based spectrometer. In conclusion, everything mentioned above contribute to reducing the size and cost of the spectrometer. The approach proposed in this paper has a bright application prospect in fabricating the practical miniature spectrometer.

Our future work is mainly focused on improving the algorithm and designing the compact hardware. Since the reconstruction results above are still slightly discrepant from the true spectra, it is quite necessary to modify the algorithm used in this paper. Moreover, it is necessary to find out the relationship between the number of filters and the needed properties of the transmission functions. As regards the hardware implementation, we try to design a compact and portable miniature spectrometer with a proper filter array attached to the photodetector [16,17,18,19,20]. It is undeniable that this is an applicable and efficient method of miniaturizing the spectrometer. Furthermore, we also attempt to implement an imaging spectrometer using a rotational filter array. 

## Figures and Tables

**Figure A1:**
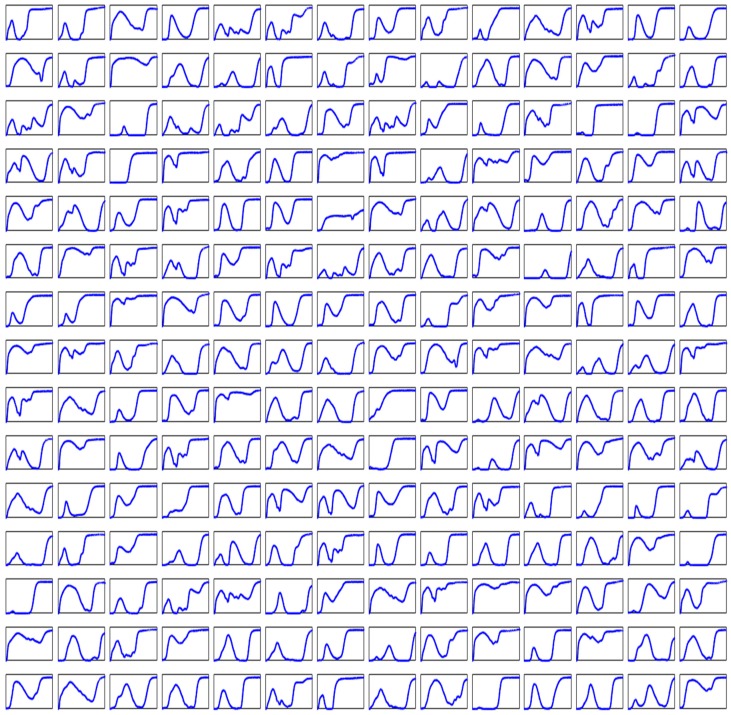
The transmission functions of the 210 filters used in the experiments. The horizontal axis is the wavelength (nm) ranging from 300 nm to 800 nm, the vertical axis represents the transmittance.

## Appendix B

Besides the optimization problem (5), there are several other forms of the *l*_1_-norm minimization to solve the inverse problem (2). Herein, we mainly introduce the following three versions briefly.

The first one is the classical Lasso problem [42],

(A1)
$$\min_{x}\;{\left\|\Phi x-b\right\|}_{2}\;s.t.\;{\left\|x\right\|}_{1}\leq \eta$$

where η is the sparse constraint on x .

Based on the Lasso problem, we can use the regularized version of (A1) as follows,

(A2)
$$\min_{x}\;{\left\|\Phi x-b\right\|}_{2}^{2}+\lambda {\left\|x\right\|}_{1}$$

where λ is the regularization parameter. Besides, the approach can be called the *l*_1_-regularized least squares [35].

The last is a more complicated one using the smoothing technology, which can be tackled by the TFOCS solver [36,37],

(A3)
$$\;\min_{x}\;{\left\|x\right\|}_{1}+\frac{1}{2}\mu {\left\|x-x_{0}\right\|}_{2}^{2}\;s.t.\;{\left\|\Phi x-b\right\|}_{2}\leq \delta$$

where μ is the regularization parameter and δ is the threshold of the noise energy.

If the parameters of the aforementioned versions are properly adjusted, the results of them will be consistent with each other. Various solvers such as CVX [34], *l*_1_-LS [35] and TFOCS [36,37] are available to optimize these problems.
